# Paleogenomics in a Temperate Environment: Shotgun Sequencing from an Extinct Mediterranean Caprine

**DOI:** 10.1371/journal.pone.0005670

**Published:** 2009-05-22

**Authors:** Oscar Ramírez, Elena Gigli, Pere Bover, Josep Antoni Alcover, Jaume Bertranpetit, Jose Castresana, Carles Lalueza-Fox

**Affiliations:** 1 Institute of Evolutionary Biology (UPF-CSIC), Barcelona, Spain; 2 Institut Mediterrani d'Estudis Avançats (CSIC-UIB), Esporles, Mallorca, Spain; Max Planck Institute for Evolutionary Anthropology, Germany

## Abstract

**Background:**

Numerous endemic mammals, including dwarf elephants, goats, hippos and deers, evolved in isolation in the Mediterranean islands during the Pliocene and Pleistocene. Most of them subsequently became extinct during the Holocene. Recently developed high-throughput sequencing technologies could provide a unique tool for retrieving genomic data from these extinct species, making it possible to study their evolutionary history and the genetic bases underlying their particular, sometimes unique, adaptations.

**Methodology/Principals Findings:**

A DNA extraction of a ∼6,000 year-old bone sample from an extinct caprine (*Myotragus balearicus*) from the Balearic Islands in the Western Mediterranean, has been subjected to shotgun sequencing with the GS FLX 454 platform. Only 0.27% of the resulting sequences, identified from alignments with the cow genome and comprising 15,832 nucleotides, with an average length of 60 nucleotides, proved to be endogenous.

**Conclusions:**

A phylogenetic tree generated with *Myotragus* sequences and those from other artiodactyls displays an identical topology to that generated from mitochondrial DNA data. Despite being in an unfavourable thermal environment, which explains the low yield of endogenous sequences, our study demonstrates that it is possible to obtain genomic data from extinct species from temperate regions.

## Introduction

Paleogenomics, the study of genomes from extinct organisms, is an emerging scientific field that has been fuelled by recently developed technologies in high-throughput DNA sequencing [1], [2]. In the first of such approaches to be undertaken [3], about 27,000 base pairs (bp) of cave bear (*Ursus spelaeus*) genomic sequences were obtained with cloning vectors from 42,000 and 44,000 years-old cave bear samples, respectively. Using sequencing-by-synthesis (SBS) technology, as applied through the Roche/454 Life Sciences GS20 and FLX pyrosequencing platforms [4], 13 million bp of the woolly mammoth (*Mammuthus primigenius*) genome were generated from a 28,000-year-old permafrost mammoth bone [5], complete mitochondrial genomes from mammoth and thylacine hairs [6], [7] and, finally, about 80% of the nuclear genome from ∼20,000-year-old mammoth hairs [8]. The same approach has been applied to other ancient bones, including Neanderthal samples that provided around 1 million bp of its genome [9], as well as other Pleistocene mammals from Denisova cave in Siberia [10]. However, the efficiency of these metagenomic analyses is notably variable: while in the mammoth bone it was possible to identify from 45.4% (mainly in bone samples) to 90.45% (in hair samples) of the sequences as endogenous, this fraction was significantly reduced in cave bear (between 1.1 and 5.8%), Neanderthal (6%, although a significant fraction of contamination was posteriorly estimated to be present in this particular extract [11]), ancient horse (0.7%), ancient wolf (1.8%) and cattle (1.1%) [3], [5], [9], [10]. In addition, due to the low genomic coverage, the degradation of the template DNA, and the innate error rate of the sequencing platforms [12], the paleogenomic data contains a significant number of sequencing errors, resulting in an excess of C to T substitutions due to cytosine deaminations as compared to the corresponding reference genome [3], [5], [9], [13]. Thus, it is likely that in the future, specific loci in regions with low shotgun coverage would need to be verified by targeted approaches, such as the polymerase chain reaction (PCR). Several studies in mammoths and Neanderthals have already focussed on the specific retrieval of nuclear genes and the problems of distinguishing endogenous variants from DNA damage [14]–[16].

Paleogenomic data can be useful for understanding the rate and nature of some evolutionary processes, because it allows us to investigate the genetic basis of adaptive traits in extinct species [14]. At present, however, it is not clear what the limits of these new technical approaches are, in terms of efficiency (ratio of endogenous versus exogenous DNA retrieved), age of the sample, geographic location and/or thermal history. For instance, some mammal species, including goats, cervids, elephants and hippos, have gone extinct in the last few thousands years in the Mediterranean islands [17 and references therein], a temperate area which is clearly not favourable to DNA preservation. The possibility of having access to the genome of these species is therefore of great interest for exploring unique insular evolutionary patterns.

In previous studies [18]–[20], we have retrieved by PCR mitochondrial genes (Cytochrome *b*, 12S rRNA) and a multi-copy nuclear gene (28S rRNA) from one of these species, *Myotragus balearicus*, an extinct goat from the Western Balearic Islands (Western Mediterranean). *Myotragus* is an extremely modified caprine [21] that evolved in insularity conditions since the end of the Messinian crisis (5.35 million years ago) in the islands of Mallorca and Menorca [22], [23]. It became extinct between 3,700 and 2,040 years BC, probably after the arrival of modern humans to the Balearic Islands [24], that took place between 2,350 and 2,150 years BC [25]. The unclear taxonomic position of this caprine is related to its amazing morphological peculiarities, which include extreme size reduction (250–500 mm shoulder height), a single, ever-growing rodent-like lower incisor, shortened distal limb bones, frontal eyes, and reduced brain size [26]–[31]. Although ancient mitochondrial DNA (mtDNA) data have provided statistical support for a *Myotragus* clade with *Ovis* [20], the general phylogeny of the caprine group is not yet fully established [20]. Here, we demonstrate that it is possible to undertake shotgun sequencing approaches from ancient bones from the thermally unfavourable Mediterranean area. Additionally, the paleogenomic data obtained from *Myotragus* support phylogenetic relationships previously generated with mtDNA sequences.

## Results

A total of 96,357 singleton GS-FLX sequence reads were obtained and analysed by means of database searches. No significant identity was found for 98.49% of the sequences, a figure higher than that found in the ancient wolf, horse and cattle (86.8% on average), Neanderthal (79%) and mammoth shotgun (5.53%, 18.4% and 24.92%, depending on the study) [5], [8], [9], [10]. A fraction of those sequences could be endogenous, but remain unidentified due to the incompleteness of the cow and specially of the sheep genome. Alternatively, the high fraction of sequences without any match may reflect a lack of environmental DNA studies in the Mediterranean area. The remaining 1.51% of the sequences were taxonomically classified by the highest identity found in the database.

**Figure 1:**
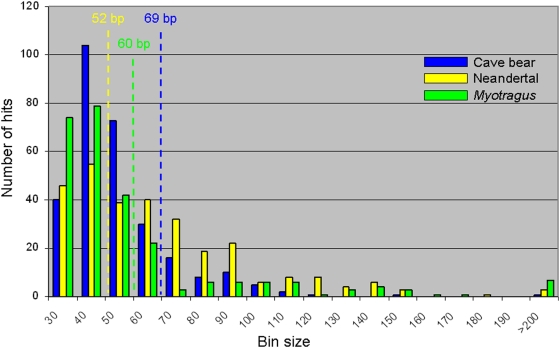
Size distribution, plotted in 10 bp bins, of Neandertal [10], cave bear [3] and *Myotragus* sequences obtained from metagenomic analyses. The average hit size in each case is indicated by a dotted line.

Only 0.27% of the sequences, comprising 15,832 nucleotides, gave the best hit to the cow genome, with an average percentage identity of 94.95%. This figure seems to be in agreement with divergence times of about 12–14.3 million years for the cattle-caprine lineages, as suggested from genetic and morphological data [32]. In addition, 0.35% of the sequences gave the best hit to the human genome, with almost 100% of identity, indicative of exogenous contamination. The most represented taxonomic group, however, was bacteria (0.69%), followed in decreasing order by invertebrates (0.12%), plants (0.05%), fungi (0.02%) environmental sequences (0.02%) and others (0.02%). The average length of these sequences was 59.97 nucleotides, and they ranged from 30 bp (determined by the length cut-off in the analysis) to 245 bp (limited by the GS-FLX technology) (Figure 1). The length was similar to those of putatively endogenous sequences found in the Neanderthal and the cave bear metagenomic library (52 and 69 nucleotides, respectively) [3], [33]. The presence of two sequences deriving from the *Bos* Y chromosome indicated that the *Myotragus* specimen analysed was likely male.

The human contaminant sequences were significantly longer on average than the *Myotragus* ones (81.57 and 59.97 respectively, *P*<0.0001), suggesting that they were more recent and therefore, less degraded. The longest (>200 bp) *Myotragus* sequences did not have higher identities to the cow sequences than the shorter ones (85.6% versus 94.95%, respectively), indicating that they are unlikely to derive from recent cow-mediated DNA contamination. Furthermore, no remains of cow were found inside the Cova Estreta cave.

To additionally confirm that the bovid-like DNA fragments were endogenous, we designed five primer pairs from the shotgun sequences that matched *Myotragus* specific substitutions in their 3′ ends and represented unambiguous (those that did produce only one match to the *Bos* genome) BLAST hits. These nuclear fragments, varying between 80 and 112 nucleotides in length, were co-amplified with a previously known 113 bp fragment of the 12S mtDNA gene [20]. In the PCR, we used, to overcome inhbitors present in ancient extracts, rat serum albumin (RSA) [34] instead of the usual bovine serum albumin (BSA) to avoid possible cow contamination in the BSA. One nuclear fragment, along with the mtDNA gene, showed an amplification product and was subsequently cloned and sequenced. The nuclear sequence was identical to that obtained in the shotgun sequencing except for two nucleotide changes that could be related to DNA damage, both in the shotgun and in the PCR-generated sequence (Figure S1).

**Figure 2:**
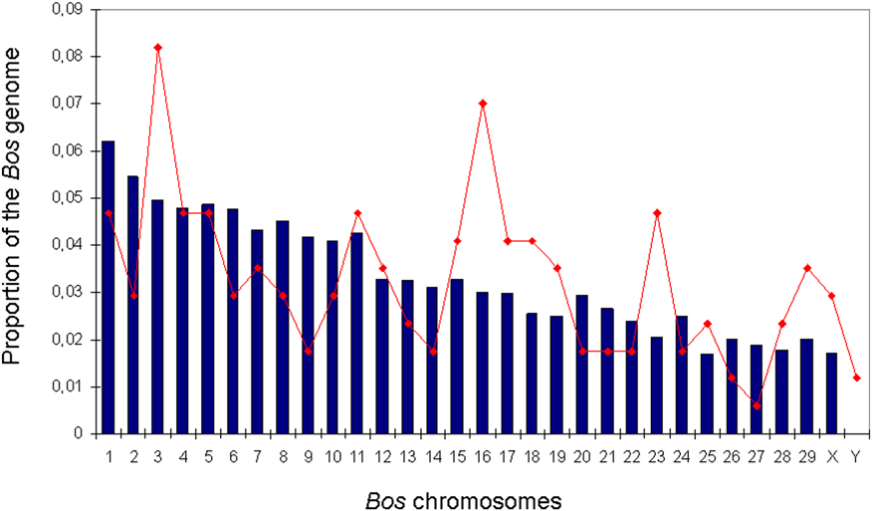
The proportion of the *Bos* genome contained on each chromosome (blue bars) is shown with the proportion of *Myotragus* sequences (red line) aligning to each *Bos* chromosome with exactly 1 hit with e-value<1e-3 BLAST. The observed distribution is not significantly different from the expected one when we compare all the chromosomes together (*P* = 0.081) or when we tested each one independently and correct for multiple testing.

The plotting of the *Myotragus* sequences along the *Bos* chromosomes showed an excess of sequences in chromosomes 3, 16 and 23, although they were not statistically significant after applying a Bonferroni correction (Figure 2). This pattern could correspond to chromosomal duplications unique to the *Myotragus* lineage or shared by all the Caprinae species, although more sequences and the completion of the *Ovis* genome are needed to explore in the future this possibility. Most of the identified *Myotragus* sequences correspond to unannotated genomic regions of the cow, with only 3.42% of the sequences and 3.90% of the nucleotides belonging to coding regions (Figure S2). The predicted *Myotragus* genes are listed in Table S1.

**Figure 3:**
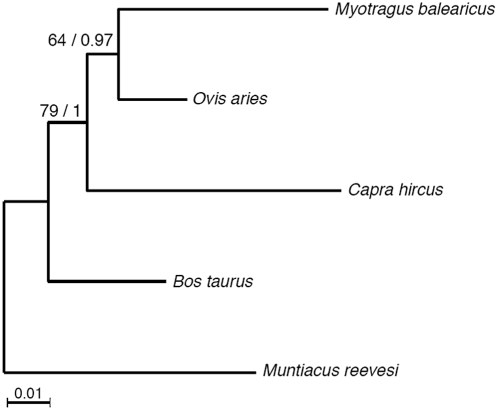
Maximum-likelihood phylogenetic tree of *Myotragus balearicus* and other artiodactyls. The tree was rooted in the cervid *Muntiacus reevesi*. Numbers along the branches indicate bootstrap support of the maximum-likelihood analyses (first number) and Bayesian support of an independent Bayesian analysis (second number). The scale bar represents 0.01 substitutions/site.

To explore the phylogenetic signal of the *Myotragus* sequences, we further searched for orthologous sequences in three Bovidae species (*Bos taurus*, *Ovis aries* and *Capra hircus*) and one Cervidae species (*Muntiacus*) in GenBank. However, we noticed a greater genomic coverage of the *Bos* genome that generated an excess of matches due to the presence of multiple paralogs. These sequences might remain undetected in the other genomes due to their more limited coverage. Therefore, we created a sub-dataset of 80 sequences (accounting for a total of 1,987 nucleotides after removing gaps and missing data) that included only those sequences that did not produce multiple matches in none of the genomes. With these sequences, we generated a maximum-likelihood phylogenetic tree that showed the topology previously established from mtDNA data for these species [20] (Figure 3), in which *Myotragus* grouped first with *Ovis*. However, the bootstrap support for this tree was low (64%). The same topology was found with Bayesian trees with a probability of 0.97 for the *Myotragus*-*Ovis* group. The overall congruence of this partial genomic phylogeny and the mtDNA tree further supports the authenticity of the *Myotragus* sequences.

**Figure 4:**
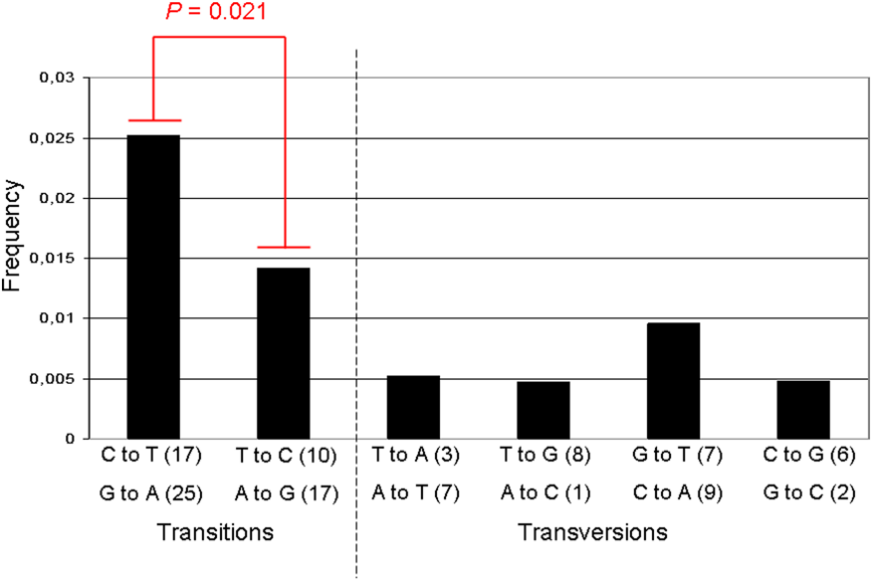
Frequency distribution of 113 *Myotragus*-specific substitutions observed in 3,602 bp of aligned *Ovis*, *Myotragus* and *Bos* genomic sequences. Complementary substitutions (such as C to T and G to A) are considered equivalent events. Fisher's exact test was used to calculate the excess of *Myotragus*-specific C to T and G to A transitions. The total number of each substitution is in parentheses.

The large branch found for *Myotragus* in the phylogenetic tree (Figure 3) could be attributed to sequence changes due to DNA damage or to an accelerated evolution of the *Myotragus* genome. To test these possibilities, we characterised the nucleotide changes exclusively present in the aligned *Myotragus* sequences (and different to those from *Ovis* and *Bos*) and found a statiscally significant (*P*<0.05) bias towards higher C to T/G to A ratios, as compared to the T to C/A to G (Figure 4, Table S1). This feature has been previously described as damage-derived lesions due to cytosine deaminations [35]. However, the removal of these substitutions from the alignment only barely shortened the *Myotragus* branch in subsequently generated trees. Specifically, the *Myotragus* branch was 3.1 times longer than the *Ovis* branch in the original alignment and it was still 2.8 times longer than *Ovis* after removing putatively damaged positions. Thus, an important contribution of accelerated evolution in the *Myotragus* genome cannot be discarded. However, a similar acceleration in *Capra* indicates that this phenomenon is not specific to the Balearic lineage.

## Discussion

Molecular studies, mainly based on mtDNA data, have failed until now to fully resolve the caprine phylogeny, probably due to the explosive radiation of this group [20]. The phylogenetic analysis of the present paleogenomic data supports the previous caprine relationships established from mtDNA, but also indicates the potential of this approach for testing evolutionary hypothesis and establishing robust phylogenies.

Despite being excavated in a region with a mean annual temperature of 14°C and below 40 degrees North latitude, we have been able to successfully retrieve nuclear genome sequences from a ∼6,000 years old *Myotragus balearicus* bone. The extremely low efficiency of the paleogenomic retrieval is striking, as is the fact that the level of human contaminant sequences is higher than that of the endogenous ones (0.34 vs 0.27). In contrast, the ratio of endogenous to contaminant human sequences among the colder preserved Denisova mammalian samples was 49∶1 [10], and the human sequences accounted for less than 0.015% [10]. In a mammoth sample from the Artic Circle [5], this ratio was 32∶1, and the human contaminants up to 1.4% of the total sequences. The *Myotragus* sample was retrieved with no special precautions against contamination. However, the histological structure of the bone also correlates with contamination levels [36], and the cortical tissue in *Myotragus* limb bones is thinner than in other, larger extinct mammals, such as mammoths and Neanderthals. Thus, it is not clear if this figure can be taken as an estimate of potential human contamination in ancient bone specimens stored in museums.

The efficiency ratio of retrieval of endogenous *Myotragus* sequences is the lowest among those observed in some other bone-based metagenomic studies, obtained from samples at higher latitudes: 47.4°N [3], 74°N [5], 45.5°N [8] and 51.23°N [10]. Despite the low efficiency values, the mean fragment length and the range value of the *Myotragus* sequences are similar to those found in samples with higher efficiencies. In addition, the frequency of damage-derived lesions in the *Myotragus* sequences is 4.2 times lower than those found in Neanderthal sequences [33]. Even so, some inconsistencies have been found between the shotgun and the PCR-based sequence, indicating the need for targeted approaches in genomic regions with low coverage. A previous study has estimated that a 12-fold coverage would be needed to have an error rate of 1 in 10,000 nucleotides [37], something extremely expensive to achieve in highly degraded ancient samples.

These somehow contradictory results between low retrieval efficiency and low DNA damage can be due to a combination of factors. On one hand, the temperate climatic conditions of the Mediterranean islands are highly unfavourable to paleogenomic preservation, although the cave where the bones were found has maintained a rather constant temperature inside. On the other hand, the *Myotragus* sample used is much more recent than those from wolf, horse, cattle, cave bear, Neanderthal and mammoth, all of them dated between 20,000 and 69,000 years ago [3], [5], [8], [9], [10]. However, the estimated thermal age [38] for this bone at the excavation is 26,206 years at 10°C (David Harker, personal communication). This age is older than that estimated for the 100,000 years-old Scladina Neanderthal [38], which is in agreement with the low efficiency of DNA retrieval found in the present study.

Our findings imply that we are working at the very limits of the current paleogenomic approaches, but still they are more efficient than PCR-based strategies, which are problematic for genomic studies on similarly preserved samples. In fact, under these unfavourable environmental conditions, only paleogenomic approaches can provide the amount of sequence data generated here. In the future, with greater genomic coverage, paleogenomic approaches could provide further data to study other aspects of this Balearic endemism, such as evidences for selective sweeps in the *Myotragus* genome related to its particular adaptations. Also, our results suggest that livestock domestication events that took place in the Fertile Crescent could be approachable from paleogenomics.

## Materials and Methods

A left *Myotragus* radius bone (IMEDEA 43619) from Cova Estreta (Pollença, Mallorca) was chosen for analysis because of its excellent macroscopic preservation. Previous analyzed bones were excavated in a different site, Cova des Gorgs (Escorca, Mallorca) [20]. Cova Estreta is a deep and narrow cave discovered in 1996 that acted as a natural trap for *Myotragus* [31]. Radiocarbon dates from bones obtained from the same stratigraphical unit [UtC-5175, 6,357±44 BP (5469–5225 calBC)] and [UtC-5171, 5,720±60 (4716–4449 calBC)] allow us to establish a narrow chronological age for the studied material of ∼6,000 years.

A sample of 3 g of cortical tissue was powdered, digested with proteinase K and extracted with phenol-chlorophorm, following a protocol described elsewhere [5]. Previous metagenomic studies have described an overwhelming fraction of environmental DNA found in ancient bones. Following a previously published procedure, the bone powder was incubated with bleach for five minutes, prior to extraction [39]. It was assumed that this could remove part of the pervasive exogenous DNA and thus increase the efficiency of the endogenous DNA retrieval. The fact that so much contamination is still seen afterwards is intriguing. Further studies could help clarify the efficiency of the bleach treatment prior to GS-FLX 454 sequencing.

One hundred microliters of extract were subjected to the GS-FLX 454 sequencing platform. The nebulization and Ampur purification steps were omited for the library building process, following, except for this, the manufacturer's guidelines (Roche Diagnostics). The amount of DNA in the libraries was estimated by Quantitative PCR (qPCR) [40] and found to be too low for successful sequencing. Therefore, libraries were amplified with the emulsion primers prior to the emulsion PCR (ePCR) to increase the amount of DNA. This procedure generated redundant sequences that were posteriorly identified and eliminated. Subsequently half of a full sequencing run was performed on the commercial Cogenics Genome Express FLX platform (Grenoble, France). To confirm the authenticity and accuracy of the GS-FLX generated data, a small number of mtDNA and nuclear DNA sequences were targeted using conventional PCR protocols, following a two-steps protocol [41] and 50 degrees of annealing temperature. Amplification products were cloned using the TOPO TA cloning kit (Invitrogen), and sequenced using an ABI3730 capillary sequencer (Applied Biosystems).

Obtained sequences were identified with BLAST searches [42] (using the megaBLAST program with an e-value threshold of 0.001) using the cow and human genomes, the environmental sample sequences database in the GenBank *env*, and the general nucleotide sequences *nt*. Sequences of other bovids (*Bos taurus*, *Ovis aries* and *Capra hircus*) as well as one cervid (*Muntiacus reevesi*) were aligned to those of *Myotragus* with Multialin [43]. Discrepancies in homopolymeric tracts were not considered, as 454 technology is known to have problems dealing with these regions [33]. Best match to target sequences in the blast didn't include the edge nucleotides, since these are known to accumulate postmortem damages associated to the breakage DNA process.

Phylogenetic trees were constructed by maximum likelihood with the Phyml program, version 2.4.4 [44]. A general time reversible (GTR) model with four rate categories and a proportion of invariable sites was used, with parameters estimated from the data. A bootstrap analysis with 100 replicas was also performed. In addition, a Bayesian tree was calculated with MrBayes 3.1 [45] using a GTR model with invariable sites and rate heterogeneity. Two runs of four chains of 5,000,000 trees were generated, sampling every 100 trees, with burning completed by the 20,000th tree.
